# Emerging hazard effects of proton pump inhibitor on the risk of colorectal cancer in low-risk populations: A Korean nationwide prospective cohort study

**DOI:** 10.1371/journal.pone.0189114

**Published:** 2017-12-07

**Authors:** In Cheol Hwang, Jooyoung Chang, Sang Min Park

**Affiliations:** 1 Department of Family Medicine, Gachon University Gil Medical Center, Incheon, Republic of Korea; 2 Department of Medicine, Seoul National University College of Medicine, Seoul, Republic of Korea; 3 Department of Family Medicine, Seoul National University College of Medicine, Seoul, Republic of Korea; 4 Department of Biomedical Sciences, Seoul National University College of Medicine, Seoul, Republic of Korea; Kagoshima University Graduate School of Medical and Dental Sciences, JAPAN

## Abstract

**Purpose:**

Despite plausible mechanisms, the clinical significance of long-term proton pump inhibitor (PPI) use to colorectal cancer (CRC) remains unknown. The purpose of this study was to investigate the association between PPI use and CRC development.

**Methods:**

We conducted a population-based prospective cohort study using the Korean nationwide claims database merged with national health examination data. The study cohort included a total of 451,284 participants who were tracked to identify cases of CRC since 2007. We assessed and standardized PPI use before the index date using the Defined Daily Dose system. We calculated the hazard ratios and their 95% confidence intervals to assess the association between PPI use and CRC occurrence using Cox proportional hazard regression models with adjustment for potential confounders. We performed subgroup analyses of the effect of PPI exposure on CRC development stratified by the CRC risk.

**Results:**

There were 5,304 cases of CRC during the study period of 2,908,152 person-years. PPI use was not associated with CRC risk overall. The incidence of CRC was higher among individuals who were elderly, male, more obese, and drank alcohol more frequently and among those who had more comorbidities. Further subgroup analyses revealed that the hazard effect of PPI use increased linearly in a dose-dependent manner with the number of CRC risk factors for which the risk level was considered low.

**Conclusion:**

Within the low-risk population, PPI use was associated with an increased risk of CRC, although the association did not weigh the effects of conventional risk factors.

## Introduction

Proton pump inhibitor (PPI) is the mainstay treatment for acid-related diseases such as peptic ulcer disease and gastroesophageal reflux disease [1]. PPI is frequently prescribed to patients with functional dyspepsia and is also prescribed for prophylactic use against non-steroidal anti-inflammatory drugs. Although for most indications PPI should only be used for 4–8 weeks, many patients require long-term use [2]. There are concerns about the safety of long-term PPI use, with increasing concern for a possible association with the risk of gastrointestinal cancers including colorectal cancer (CRC) [3, 4].

CRC has long been considered a Western disease, but the incidence of CRC has been steeply increasing in the Asia-Pacific region during past decades [5]. Korea had the highest age-standardized incidence worldwide (45.0 per 100,000 individuals) according to the GLOBOCAN 2012 because of an exponential increase in obesity and a decrease in physical activity, which result from a Westernized lifestyle accompanied by a marked increase in the consumption of calorie-rich diets [6]. CRC represents a large economic burden in Korea [7]. Hence, efforts to control risk factors as well as to detect CRC earlier are of substantial importance.

One novel mechanism by which PPI increases CRC risk is through the hormone gastrin. Beyond its role in gastric acid secretion, gastrin is known to stimulate the growth of epithelial cells and to prevent apoptosis [8]. The normal feedback process between gastric acid and serum gastrin could lead to a chronic state of hypergastrinemia (i.e., PPI-induced hypergastrinemia). The carcinogenicity of PPI in the lower gastrointestinal tract has been studied extensively in animals. Chronic hypergastrinemia was shown to promote the growth of malignant colonic epithelial cells; conversely, gastrin antagonism inhibits the growth of CRC [9, 10].

The relevance of the previous findings to humans is less clear. A meta-analysis including five case-control studies reported a non-significant increase in CRC risk among PPI users [11]. A recent nested case-control study demonstrated that PPI users had a 2.5-fold higher risk of CRC development (95% confidence interval [CI], 2.31–2.79) compared with non-users [12]. However, the mean duration of PPI exposure in that study was just 75 days prior to the diagnosis of CRC, and the result was interpreted as an indication that PPI use can be helpful in the early detection of occult CRC.

The previous epidemiological studies had several important limitations. One study [13] included patients with Barrett’s esophagus and matched controls; two studies [13, 14] lacked the power to detect differences in CRC risk; and several risk factors for CRC were not available for analysis, such as comorbidities [14, 15] (especially type 2 diabetes) and lifestyle habits (e.g., smoking [15], alcohol consumption [14], and obesity [15]). No study has been performed to examine the differential effects of PPI exposure on CRC development in groups with various known risk levels.

Researchers have sought, although all have failed, to explain the inconsistency between the experimental results and the clinical evidence by enlarging the sample size [12, 15, 16], lengthening the duration of follow up [14–16], or analyzing more potent exposures to PPI [15–17]. A newly conceived risk factor is not necessarily independent of all conventional ones, especially if the conventional risk factors strongly impact on the outcome. On such occasions, stratified analyses across the risk levels might be a better alternative. To understand the implications on both clinical practice and further research, it is critical to determine whether the association is merely insignificant or truly non-significant.

Here, we investigated the association between PPI exposure and CRC development in an area where CRC is prevalent. In addition, regardless of the overall association, we performed a further analysis to determine whether the association varies according to CRC risk factors. In contrast to previous nested case-control studies on this issue, we prospectively designed a cohort observation to ensure equity of exposures to risk factors other than PPI use.

## Materials and methods

### Data source and study design

We used the data of a standardized cohort, which were provided for research purpose by the Korean National Health Insurance Corporation (NHIC) with strict confidentiality guidelines. We merged the data from the NHIC claims database with those from national health examination, which the NHIC provides biennially to all dependents over 40 years of age.

The Korean NHIC is the single insurer of the Korean public health-insurance sector, providing compulsory universal health insurance covering nearly all forms of health care services. From the original cohort data, we extracted the following information about individuals: sex, date of birth, average monthly insurance premium, details on admissions and outpatient visits, comorbid conditions, and prescription data including drug name, dosage, duration, and total expenditure. We verified that patients actually received the drugs prescribed by cross checking pharmacy visits. We selected the health examination nearest to the index date (January 1, 2007) and extracted the following information: height, weight, and self-reported health-related habits (tobacco use, alcohol consumption, and physical activity). The NHIC databases have been used for epidemiologic research with high validity in the past [18].

**Fig 1:**
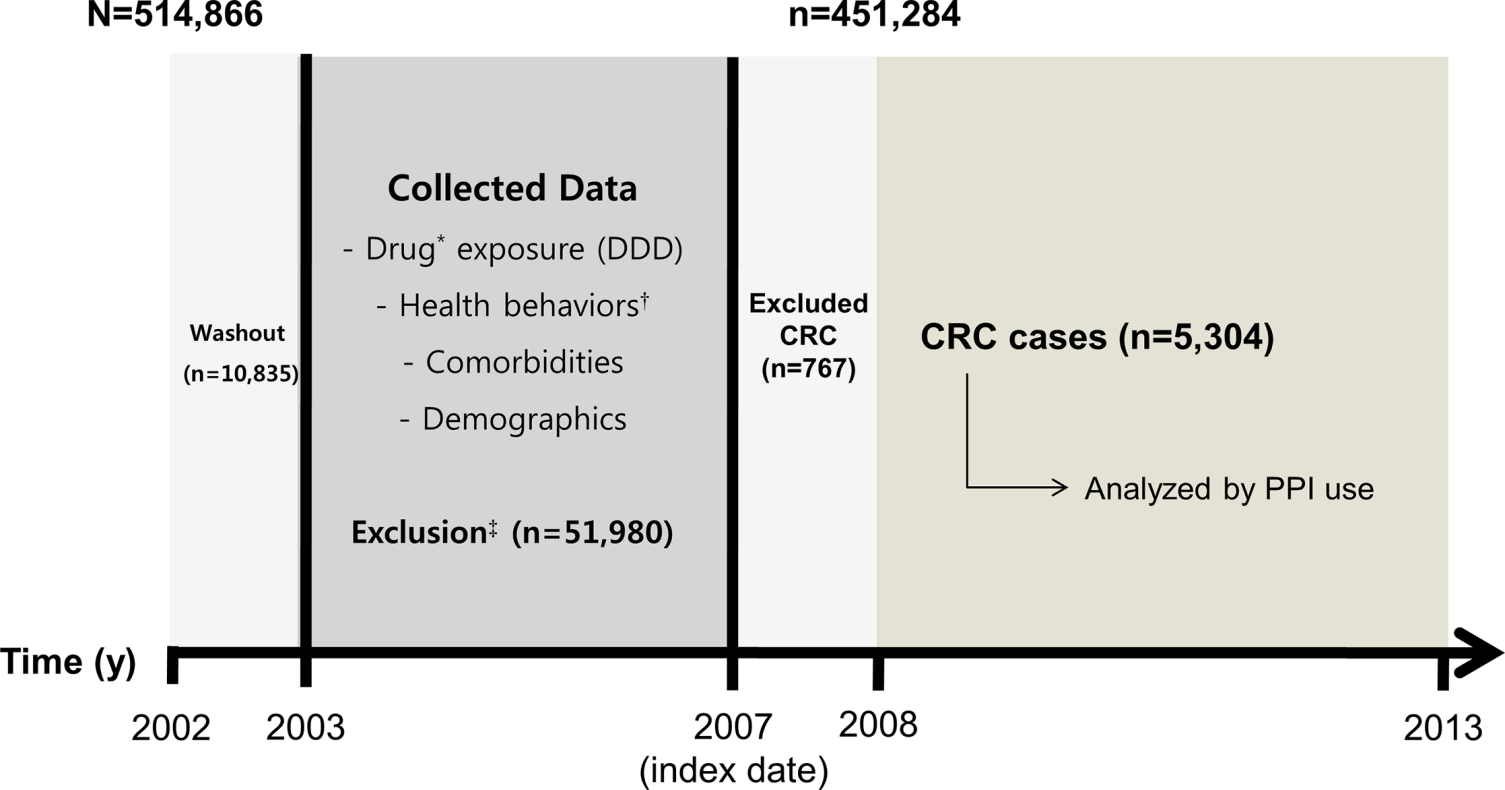
Study design and recruitment of participants. DDD, defined daily dose; CRC, Colorectal cancer; PPI, proton pump inhibitor; NHIC, National Health Insurance Corporation. *Using the claims database of the NHIC including aspirin, statin, and metformin. ^†^From national health examinations including smoking status, drinking habit, and physical activity. ^‡^Patients with any cancer diagnosis by ICD-10 “C” code, with past medical history of cancer according to health-check survey data, who died before the index date, or had missing non-survey health check-up variables were excluded from study.

From the NHIC records, we identified individuals who were 40 years of age or older and used the Korean national health examination service at least once between January 1, 2002 and December 31, 2006 (N = 514,866). To minimize the effect of PPI use prior to the study period, we excluded the individuals who were prescribed PPI in 2002 (n = 10,835). Participants were ineligible for the study (n = 51,980) if they had a medical history of cancer according to health check-up survey data, died before the index date, were missing any non-survey health check-up variables, or had been diagnosed with any cancer, indicated by the *International Classification of Diseases code–10th Revision* (ICD-10) “C” codes, before December 31, 2006. To minimize a protopathic bias resulting from a failure to detect palliative PPI use by patients with CRC, we also excluded participants who were diagnosed with CRC within 1 year after the index date (n = 767). Finally, we included a total of 451,284 participants in the analysis (Fig 1). The ethics committee waived the requirement for participant consent, and the Institutional Review Board of Seoul National University Hospital (IRB number: E-1509-004-699) approved the study protocol.

### Assessment of exposures and covariates

We extracted the information about all exposures and covariates during the 5 years prior to the index date. The primary exposure of interest was the cumulative use of PPI. We collected data pertaining to PPI prescriptions such as the dates of prescriptions, the daily dose, the number of days supplied, and the number of pills per prescription. To indicate the PPI exposure, we used the Daily Defined Dose (DDD) system. We computed the cumulative daily dose (in units of DDD) and categorized the PPI users into three groups based on the cut-off values of 60 DDDs and 180 DDDs. We used similar processes to assess the use of drugs that may influence the risk of CRC: aspirin, statins, and metformin. Patients who consumed less than 60 DDDs of those drugs were defined as non-users.

We expressed comorbid conditions as a Charlson comorbidity index (CCI) score, which was derived from the ICD-10 codes in the claims database. We calculated the CCI using the sum of the weighted scores of all comorbidities excluding diabetes (e.g., cardiovascular diseases, pulmonary diseases, renal diseases, liver diseases, and other diseases) [19]. We calculated body mass index (BMI) as the weight divided by the square of the adult height (kg/m^2^). For analysis, we classified the participants based on the following categories: BMI (<23.0, 23–24.9, 25–29.9, or ≥30 kg/m^2^), frequency of physical activity (none, 1–2, or ≥3 times/week), smoking status (never, former, or current smoker), frequency of alcohol consumption (none, 1–2, or ≥3 times/week), CCI score (0, 1–2, or ≥3), and socioeconomic status (quartiles 1–2 [low] or quartiles 3–4 [high]).

### Ascertainment of incident CRCs

The primary outcome was a new diagnosis of CRC, represented by the ICD-10 codes in the nationwide claims database, during the observation period. We defined CRC cases as those in which patients who visited the hospital at least once and received ICD-10 code “C18”, “C19”, or “C20” met any of the following additional criteria: (i) made at least three outpatient visits related to the code, (ii) had 3 or more days of admission with the code, (iii) received any curative cancer treatments claimed via the Korean Diagnosis-Related Group (HDRG) code “G60-Digestive Malignancy”, (iv) died of causes related to the code. In cases meeting those criteria, the first date of diagnosis under the code was defined as the date of the event. Cases that met the criteria but involved a diagnosis of any other cancer prior to the date of the event were not considered CRC cases for the purpose of our analysis. We observed the participants from the index date until December 31, 2013.

### Statistical analyses

We compared the baseline characteristics of the study population according to the level of PPI exposure using a χ^2^ test. The primary analysis was a Cox proportional hazards analysis to estimate hazards ratios (HRs) and 95% CIs for the association between PPI use and CRC risk. We calculated the accumulated person-years of risk, beginning with the index date and ending with the date of CRC diagnosis, diagnosis of any other cancer, or death or December 31, 2013, whichever came first—CRC diagnosis was the primary endpoint.

We identified the relevant factors associated with CRC risk in our cohort. We also performed subgroup analyses stratified and combined by the CRC risk factors. In the stratified multivariable analyses, we reexamined the association between PPI use and the risk of CRC in different subgroups. We performed all analyses using STATA Version 11.0 for Windows (STATA Corp., TX). *P*-values <0.05 were considered to be statistically significant.

## Results

Table 1 demonstrates the characteristics of the study population by PPI exposure levels. PPI exposure was significantly associated with all conventional risk factors (all *P*-values<0.05) such as old age, male gender, obesity, current smoking, frequent drinking, low physical activity, more comorbid conditions (including type 2 diabetes), more concurrent drug use, and low socioeconomic status.

**Table 1:** Characteristics of the study population by PPI exposure.

				Exposure level to PPI, %		
			Total cohort, %	None	<60 DDDs	≥60 DDDs
Characteristics			(N = 451,284)	(n = 401,764)	(n = 43,840)	(n = 5,680)
Age, years						
	40–49		48.4	48.8	46.6	37.4
	50–59		28.1	27.8	30.3	32.1
	≥60		23.5	23.4	23.1	30.4
Gender, male			53.5	53.4	53.7	58.6
Body mass index, kg/m^2^						
	<23.0		37.4	37.4	37.2	34.5
	23.0–24.9		27.7	27.7	28.2	27.8
	25.0–29.9		32.1	32.0	32.2	34.7
	≥30.0		2.9	2.9	2.5	2.9
Smoking status						
	Never		69.5	69.6	69.3	66.8
	Former		8.7	8.6	9.4	10.1
	Current		20.7	20.7	20.4	22.3
Alcohol consumption, drink/week						
	None		72.7	72.7	73.1	73.0
	1–2		16.2	16.3	15.8	15.2
	≥3		10.5	10.5	10.7	11.1
Physical activity, times/week						
	None		52.5	52.5	51.9	53.1
	1–2		25.4	25.4	25.6	23.9
	≥3		21.3	21.2	21.8	22.2
Comorbidities						
	Type 2 diabetes		19.7	19.1	23.3	29.7
	CCI* score					
		0	29.3	32.2	5.7	3.2
		1–2	54.5	53.1	67.2	61.2
		≥3	16.2	14.7	27.1	35.7
Concurrent drug user^†^						
	Aspirin		12.4	12.1	14.2	18.9
	Metformin		4.0	3.9	4.0	4.8
	Statin		6.8	6.5	8.6	13.4
SES, low^‡^			55.6	55.8	53.8	54.8

PPI, proton pump inhibitor; CCI, Charlson comorbidity index; SES, socioeconomic status.

*Including acute myocardial infarction, congestive heart failure, peripheral vascular disease, cerebral vascular accident, dementia, pulmonary disease, connective tissue disorder, peptic ulcer, liver disease, paraplegia, renal disease, severe liver disease, and HIV infection based on ICD-10 codes of hospital visits during years 2002 through 2006.

^†^ ≥60 DDDs.

^‡^By quartiles of insurance premium (Q1–2).

All *P*-values using a χ^2^ test were less than 0.05.

There were 5,304 cases of CRC in the total cohort during the observation period of 2,908,152 person-years. Table 2 lists the identified risk factors associated with CRC. A fully adjusted Cox proportional hazard model revealed that CRC was more likely among individuals who were elderly (HR, 1.06 per 1 year; 95% CI, 1.05–1.06), were male (HR, 1.61; 95% CI, 1.50–1.72), were more obese (*P*_trend_ = 0.043), drank alcohol more frequently (*P*_trend_<0.001), or had more comorbidities (*P*_trend_ = 0.025) including type 2 diabetes (HR, 1.14; 95% CI, 1.06–1.23). We did not find any association overall between PPI exposure and CRC risk (*P*_trend_ = 0.467).

**Table 2:** Adjusted HRs and 95% CIs for CRC associated with PPI and covariates.

		Age- and sex-adjusted			Multivariate adjusted			
		HR	95% CI		HR*	95% CI		*P*_trend_
Age (per 1 year)		—			**1.06**	**1.05**	**1.06**	
Male		—			**1.61**	**1.50**	**1.72**	
PPI exposure								
	None	1			1			0.467
	<60 DDDs	0.98	0.90	1.08	0.96	0.88	1.06	
	≥60 DDDs	1.01	0.80	1.27	0.98	0.78	1.24	
Body mass index, kg/m^2^								
	<23.0	1			1			**0.043**
	23.0–24.9	0.98	0.91	1.05	0.97	0.91	1.04	
	25.0–29.9	**1.07**	**1.00**	**1.14**	1.05	0.98	1.12	
	≥30.0	**1.23**	**1.05**	**1.43**	**1.18**	**1.00**	**1.38**	
Smoking status								
	Never	1			1			0.245
	Former	1.04	0.94	1.15	1.00	0.91	1.11	
	Current	**1.11**	**1.03**	**1.20**	1.05	0.98	1.14	
Alcohol consumption, drink/week								
	None	1			1			**<0.001**
	1–2	**1.15**	**1.07**	**1.25**	**1.15**	**1.06**	**1.24**	
	≥3	**1.36**	**1.26**	**1.48**	**1.36**	**1.25**	**1.47**	
Physical activity, time/week								
	None	1.01	0.95	1.09	1.02	0.95	1.09	0.885
	1–2	1.05	0.97	1.14	1.06	0.98	1.15	
	≥3	1			1			
Type 2 diabetes^†^								
	No	1			1			
	Yes	**1.19**	**1.12**	**1.27**	**1.14**	**1.06**	**1.23**	
CCI^‡^ score								
	0	1			1			**0.025**
	1–2	1.02	0.96	1.09	1.02	0.95	1.09	
	≥3	**1.15**	**1.06**	**1.25**	**1.11**	**1.02**	**1.22**	
Aspirin use								
	Yes^§^	**1.12**	**1.04**	**1.21**	1.07	0.99	1.16	
	No	1			1			
Metformin use								
	Yes^§^	1.25	1.11	1.40	1.12	0.98	1.27	
	No	1			1			
Statin use								
	Yes^§^	1.01	0.91	1.11	0.91	0.82	1.01	
	No	1			1			
SES								
	Low^¶^	1.04	0.99	1.10	1.03	0.98	1.09	
	High	1			1			

HR, hazard ratio; CRC, colorectal cancer; PPI, proton pump inhibitor; CI, confidence interval; CCI, Charlson comorbidity index; DDD, defined daily dose; SES, socioeconomic status.

*Using a Cox proportional hazards regression models with adjustment for all listed variables.

^†^Based on ICD-10 codes of hospital visits during years 2002 through 2006.

^‡^Including acute myocardial infarction, congestive heart failure, peripheral vascular disease, cerebral vascular accident, dementia, pulmonary disease, connective tissue disorder, peptic ulcer, liver disease, paraplegia, renal disease, severe liver disease, and HIV infection based on ICD-10 codes of hospital visits during years 2002 through 2006. Diabetes mellitus was not considered for CCI to prevent co-linearity.

^§^ ≥60 DDDs.

^¶^By quartiles of insurance premium (Q1–2).

Table 3 presents the results of subgroup analyses by the significant CRC risk factors identified in Table 2. In each of the six analyses, the hazard effect of PPI use tended to appear in the low risk population, although all effects were insignificant. We performed further subgroup analyses for combinations of the low-risk factors. Among the non-obese, non-diabetic female population who were less than 50 years of age and without a history of alcohol consumption, those who received ≥180 DDDs of PPI had a higher risk of CRC than non-users of PPI (adjusted HR = 12.30, 95% CI = 1.71–88.23; *P*_trend_<0.01). Further subgroup analyses revealed that the hazard effect of PPI exposure in that population increased linearly in a dose-dependent manner with the number of CRC risk factors for which the risk level was low. We performed a same analysis for the factors conferring a high CRC risk to check for the possibility of statistical illusion, but that analysis did not produce any statistically significant results (S1 Table).

**Table 3:** Risk of exposure to PPI for CRC development among various risk groups (reference: PPI use <60 daily defined doses [DDDs]).

				60–180 DDDs				≥ 180 DDDs			
		No. of CRC	Person-years	HR	95% CI		P*	HR	95% CI		P*
Total		5,304	2,908,152								
Age, years											
	40–49^1^	1,535	1,455,233	1.28	0.82	2.02	0.281	1.89	0.47	7.59	0.367
	≥50	3,769	1,452,919	0.91	0.69	1.22	0.537	0.67	0.25	1.79	0.428
Sex											
	Female^2^	1,983	1,369,792	1.16	0.79	1.71	0.448	1.54	0.58	4.11	0.387
	Male	3,321	1,538,360	0.92	0.68	1.26	0.606	0.46	0.11	1.82	0.266
BMI, kg/m^2^											
	<23.0^3^	1,937	1,080,021	1.21	0.83	1.75	0.326	1.88	0.70	5.02	0.207
	≥23.0	3,367	1,828,131	0.89	0.65	1.22	0.474	0.41	0.10	1.64	0.207
Drinking per week											
	No^4^	3,564	2,116,650	1.06	0.79	1.41	0.707	1.20	0.50	2.88	0.685
	Yes (1 or more)	1,706	774,431	0.88	0.56	1.37	0.570	0.36	0.05	2.55	0.306
T2D											
	No^5^	3,991	2,387,718	1.10	0.83	1.46	0.506	1.01	0.42	2.43	0.985
	Yes	1,313	520,434	0.81	0.50	1.30	0.379	0.52	0.07	3.71	0.517
CCI score											
	<3^6^	4,222	2,463,313	1.18	0.88	1.57	0.267	1.02	0.38	2.73	0.962
	≥3	1,082	444,839	0.73	0.47	1.14	0.168	0.64	0.16	2.56	0.527
*Low-risk combinations*											
	With any 6 risk factors	5,108	2,683,726	0.95	0.74	1.22	0.712	0.72	0.30	1.72	0.457
	1 & 4	932	995,601	1.66	0.98	2.83	0.060	1.59	0.22	11.32	0.643
	1 & 4 & 5	829	890,306	1.68	0.95	2.98	0.075	1.94	0.27	13.78	0.509
	1 & 4 & 5 & 3	341	381,276	**2.72**	**1.28**	**5.79**	**0.009**	6.24	0.87	44.59	0.068
	1 & 4 & 5 & 3 & 2	212	245,709	**2.84**	**1.05**	**7.70**	**0.040**	**12.30**	**1.71**	**88.23**	**0.013**
	Without any 6 risk factors (1 & 4 & 5 & 3 & 2 & 6)	196	224,425	**3.64**	**1.34**	**9.85**	**0.011**	**15.12**	**2.10**	**108.82**	**0.007**

PPI, proton pump inhibitor; CRC, colorectal cancer; DDD, defined daily dose; HR, hazard ratio; CI, confidence interval; BMI, body mass index; T2D, type 2 diabetes; CCI, Charlson comorbidity index.

*Using Cox proportional hazards regression models with adjustment for all potential confounders listed in Table 2.

## Discussion

The widespread use of PPI and the continuous rise in the incidence of CRC underlines the need for investigation of the association between PPI use and CRC. Our population-based prospective study found an association between PPI use and CRC risk in stratified sub-populations. The emerging significance in low-risk individuals suggests that the effects of PPI use on CRC risk did not weigh those of conventional risk factors on CRC development. Our results provide, to our knowledge, the first clinical evidence demonstrating a hazard effect of PPI on CRC incidence.

Despite plausible mechanisms, previous epidemiologic studies did not demonstrate any relationship between PPI use and CRC risk. Besides the stratified analysis, the design of our study differed fundamentally from those of earlier studies. In a nested-control design, a flaw derived from the process of sampling control individuals is inevitable. Controls for each case had to be at risk of CRC at the time the corresponding case was diagnosed, and they were matched principally by age [15], follow-up time [16], or both [14, 17]. The PPI exposure in each case was measured from the time of entry into the cohort until the time of the event, and that of each control was measured until the control individual reached the age at which the event occurred in the corresponding case. Thus, the follow-up time was truncated when the control individual reached the age of the index case, and other measures of exposure that depend on the follow-up time were distorted. Therefore, the nested case-control design did not allow subgroup analyses of the data by other covariates, unlike our prospective design.

In addition to the prospective design, the main strength of our study was the use of a large, Korean population-representative cohort with up to 12 years of follow-up. Very few studies of the association between the use of PPI and the incidence of CRC have been conducted in Asian countries [12], where the prescription of PPI is strictly regulated by the national health insurance system and the possibility of PPI overuse without appropriate indications is therefore very low. For the same reason, the prevalence of CRC reported here is similar to that from the cancer registry. Our study also utilized national health examination databases as well as pharmacy databases, thus decreasing the risk of misclassification for all exposure covariates. The expected effects of well-known risk factors on CRC development verified the internal validity of our dataset.

Although the deleterious effect of PPI use was confined to individuals that had a low-risk for CRC, the result is important from an epidemiologic perspective. Researchers have struggled to understand the clinical relationship between PPI use and CRC. A recent review [20] proposed two plausible explanations: (i) Gastrin has a double edged sword-like role in carcinogenesis; (ii) PPIs may directly inhibit hypergastrinemia-associated carcinogenesis. It is more convincing, however, to suppose that PPI-induced hypergastrinemia might increase the number of sporadic mutations in normal cells and/or promote the proliferation of neoplastic tissues. Among a great many experimental and animal model studies, the most direct evidence was observed in a transgenic APC genes (APC^*Min*-/+^) mouse model: omeprazole-induced hypergastrinemia resulted in a significant increase in the proliferation rate of colorectal adenomas, which was completely reversed by preimmunization [21]. A population-based study also provided strong evidence of a positive association between hypergastrinemia and CRC risk [22]. Despite some inconsistencies, the balance of the existing evidence, together with the results of our study, suggests colon-specific growth promotion by hypergastrinemia.

Several explanations have been suggested for the lack of significant results in clinical researches of the relationship between PPI exposure and CRC risk. It is uncertain whether it was due to a short duration of follow-up. The transition from normal colonic mucosa to CRC is estimated to take 10 years, so large proportions of the study populations were likely in the middle stages of the transition [23]. In addition, experimental data have shown that the adenoma-carcinoma sequence was accelerated by PPI-induced hypergastrinemia [21]. Therefore, one would expect to see an increased risk become apparent with 5 years [14–16] of follow-up time, if it truly occurs.

Another factor that might confound analyses of the relationship between PPI exposure and CRC risk is the extent of hypergastrinemia. The relationship between the level of serum gastrin and the risk of CRC is exceedingly complex. Although a modest increase in serum gastrin levels is common among long-term PPI users, a recent systematic review and two randomized controlled trials demonstrated substantial variability in the level of serum gastrin within and among individuals [24, 25]. In addition, CRC itself may induce gastrin release in an autocrine or paracrine manner [26]. Even in studies of disease entities characterized by hypergastrinemia (e.g., pernicious anemia and Zollinger-Ellison syndrome), a clinically relevant increase in CRC risk remains unclear [16, 27].

We acknowledge several limitations. First, our results cannot be generalized to other ethnicities, because the contributions of risk factors for CRC varied across the population. Many cultural and emotional elements could also be involved; for example, perceived risk (e.g., having a family history) is associated with preventive behavior such as CRC screening [28]. Second, we did not investigate the intra-class differences [29] in the association between PPI use and CRC that might arise due to individuals switching among different PPIs during treatment. Third, the NHIC database does not include clinical information on *Helicobacter pylori* status. A meta-analysis reported that *H*. *pylori* infection might be associated with a small but significantly greater risk of colorectal neoplasia [30]; the prevalence of *H*. *pylori* infection in Korea exceeds 50%. The lack of *H*. *pylori* data seemed rather to attenuate the significance, however, given that the higher proportion of individuals receiving *H*. *pylori* eradication would be higher among PPI users than among non-users.

In conclusion, our study showed an increased risk of CRC among PPI users, corroborating the results of clinical experiments. The importance of controlling for conventional CRC risk factors cannot be emphasized enough. Physicians should be aware of the association between PPI use and CRC and prescribe the lowest effective dose over the shortest possible period of time for patients with appropriate indications. More research is necessary to confirm and further characterize our findings.

## Supporting information

S1 Table High-risk combinations in the risk of exposure to PPI for CRC development (reference: PPI use <60 daily defined doses [DDDs]). PPI, proton pump inhibitor; CRC, colorectal cancer; DDD, defined daily dose; BMI, body mass index; CCI, Charlson comorbidity index; HR, hazard ratio; CI, confidence interval; NA, not applicable. All analyses for individuals receiving ≥ 180 DDDs could not be evaluated due to small sample size.
(DOC)
