# Supplementary material for: Emerging hazard effects of proton pump inhibitor on the risk of colorectal cancer in low-risk populations: A Korean nationwide prospective cohort study
Source: PLoS One. 2017 Dec 7;12(12):e0189114. doi: 10.1371/journal.pone.0189114 (PMC5720708; doi:10.1371/journal.pone.0189114)
Supplement: S1 Table — PPI, proton pump inhibitor; CRC, colorectal cancer; DDD, defined daily dose; BMI, body mass index; CCI, Charlson comorbidity index; HR, hazard ratio; CI, confidence interval; NA, not applicable. All analyses for individuals receiving ≥ 180 DDDs could not be evaluated due to small sample size. (DOC) [file pone.0189114.s001.doc]

**Supporting information**

**S1 Table.** **High-risk combinations in the risk of exposure to PPI for CRC development (reference: PPI use <60 daily defined doses [DDDs]).**

|  | | No. of CRC | Person-years | 60–180 DDDs | | | |
| --- | --- | --- | --- | --- | --- | --- | --- |
|  | |  |  | HR | 95% CI | | *P*^*^ |
| High-risk combinations | |  |  |  |  |  |  |
|  | Age≥50 & Drinker | 1,137 | 331,869 | 0.78 | 0.44 | 1.38 | 0.398 |
|  | Age≥50 & Drinker & Diabetes | 314 | 76,067 | 0.67 | 0.25 | 1.81 | 0.431 |
|  | Age≥50 & Drinker & Diabetes & BMI≥23 | 222 | 53,278 | 0.73 | 0.23 | 2.30 | 0.595 |
|  | Age≥50 & Drinker & Diabetes & BMI≥23 & Male | 203 | 46,150 | 0.53 | 0.13 | 2.13 | 0.370 |
|  | Age≥50 & Drinker & Diabetes & BMI≥23 & Male & CCI≥3 | 57 | 11,561 | NA | — | — | — |

PPI, proton pump inhibitor; CRC, colorectal cancer; DDD, defined daily dose; BMI, body mass index; CCI, Charlson comorbidity index; HR, hazard ratio; CI, confidence interval; NA, not applicable. All analyses for individuals receiving ≥ 180 DDDs could not be evaluated due to small sample size.

^*^Using Cox proportional hazards regression models with adjustment for all potential confounders listed in Table 2.
